# Complete mitochondrial genome of *Bombus consobrinus* (Hymenoptera: Apidae)

**DOI:** 10.1080/23802359.2017.1390422

**Published:** 2017-11-06

**Authors:** Xiaomeng Zhao, Jiaxing Huang, Cheng Sun, Jiandong An

**Affiliations:** Key Laboratory of Pollinating Insect Biology of the Ministry of Agriculture, Institute of Apicultural Research, Chinese Academy of Agricultural Sciences, Beijing, China

**Keywords:** *Bombus consobrinus; Megabombus;* mitochondrial genome, genetic diversity

## Abstract

The complete mitochondrial genome of *Bombus consobrinus* was sequenced. This circular mitogenome is 17,966 bp (86.7% of AT) in length, which contains 22 tRNA genes, 13 protein-coding genes and two rRNA genes. All protein-coding genes are initiated with the typical ATN codon and terminated by TAA codon except an incomplete stop codon T for *ND4* gene. *tRNA-Ser (AGN)* lacks a dihydrouridine (DHU) arm while other tRNA genes form a typical cloverleaf structure. The complete mitochondrial genome of *B. consobrinus* will be useful to understand the inter-species relationship and genetic diversity of bumblebees.

Bumblebees are important agricultural pollinators because of their unique buzz pollination (De Luca and Vallejo-Marin 2013). Nevertheless, similar colour patterns make species identification problematic (Williams 2007; Carolan et al. 2012). It has become much easier to identify species and estimate the phylogenetic relationship using mitochondrial genomic sequences (Sankoff et al. 1992; Boore and Brown 1998). *Megabombus* is one of the 15 subgenera of bumblebees all over the world (Williams et al. 2008), however, to date, no mitochondrial genome from *Megabombus* subgenus was sequenced yet. As one of the most typical species among bumblebees in the subgenus of *Megabombus*, *Bombus consobrinus* is widespread in northern China (An et al. 2014). To further the phylogenetic analysis of bumblebees, we sequenced the complete mitochondrial genome of *B. consobrinus*.

Male bumblebees of *B. consobrinus* were collected from Wulingshan nature reserve in Hebei province, China (N 40.53193; E 117.48174). Specimen is stored in the CAAS Institute of Apicultural Research, Beijing, China (IAR), accession number: IAR-CO00033. The genomic DNA was extracted from one single haploid drone, which was sequenced by Illumina’s HiSeq2500 (Illumina), with a read length of 250 bp. The resultant shotgun reads were assembled by DISCOVAR *de novo* (https://software.broadinstitute.org/software/discovar/blog/). We obtained a circular mitochondrial genome of 17,966 base pairs (bps) in length (accession number: MF995069). We analyzed the mitogenome by MITOS web server (Bernt et al. 2013), and phylogenetic analysis was performed by MEGA7 software (Kumar et al. 2016).

The complete mitogenome sequence of *B. consobrinus* consists of 13 protein-coing genes (PCGs), 22 transfer RNA (tRNA) genes and two ribosomal RNA (rRNA) genes, with an overall high level of AT content (86.7%). All PCGs distribute similarly as in other *Bombus* mitogenomes: four PCGs were located on the light strand, while others are on the heavy one (Du et al. 2016; Nishimoto et al. 2017). Both *ATP8*-*ATP6* and *ND6*-*CYTB* overlap 13 nucleotides. All PCGs use typical start and stop codons, except *ND4* with an incomplete termination codon T. Incomplete stop codon happens occasionally in bee mitochondrial sequences (Cha et al. 2007; Takahashi et al. 2016), presumably caused by mRNA polyadenylation (Ojala et al. 1981). Besides *tRNA-Ser* (*AGN*) is lack of a dihydrouridine (DHU) arm, all other tRNA genes possess a typical cloverleaf structure.

Phylogenetic relationship of mitochondrial genomes coming from 25 closely related taxa was analyzed using concatenated amino acid sequences of all 13 PCGs with maximum likelihood method (Figure 1). The phylogeny tree generated in this study suggests that species in the genus of *Bombus* exhibits a closer relationship with *Melipona* species than with *Apis* species, which is consistent with previous studies (Ascher et al. 2001; Cameron et al. 2001; Zhao et al. 2017). Within the *Bombus* clade, *B. consobrinus* is the sister group to all the other bumblebees, which coincides well with the reported simplified subgeneric phylogeny (Williams et al. 2008). Mitochondrial genome sequences will provide additional evidence for the taxonomy of bumblebee species.

**Figure 1.**
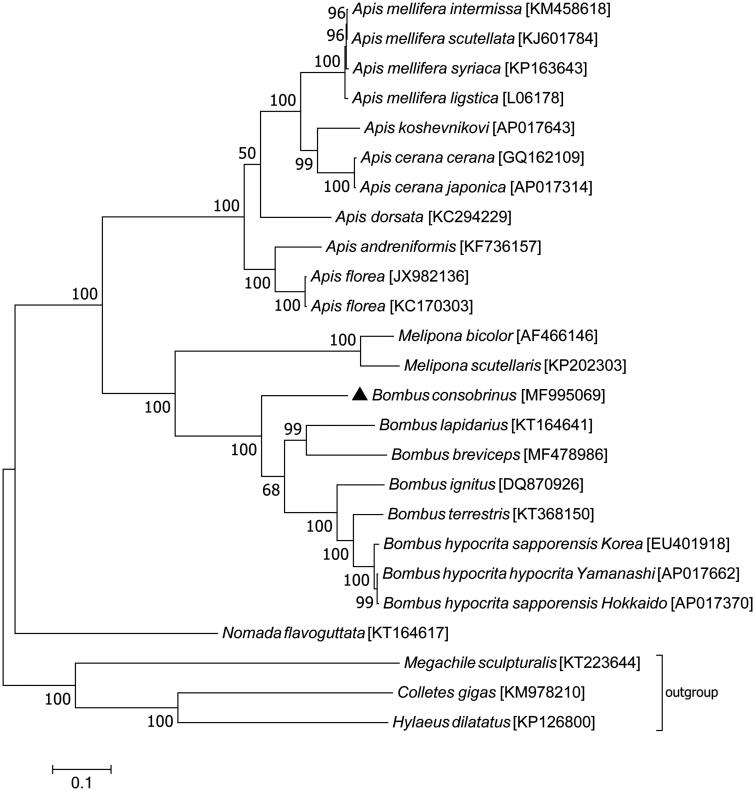
Phylogenetic analysis of the 25 related mitochondrial genomes. Black triangle indicates the focal mitochondrial genome of this study. Numbers beside each node represent percentages of 1000 bootstrap values. Mitochondrial genome sequences from Megachilidae (*Megachile sculpturalis*) and Colletidae (*Colletes gigas* and *Hylaeus dilatatus*) were used as outgroup. Species names are followed by the GenBank accession numbers of their mitochondrial genomes.
